# Meal Timing Interventions for Weight Loss and Metabolic Health—What Does the Evidence Tell Us So Far?

**DOI:** 10.1001/jamanetworkopen.2024.42140

**Published:** 2024-11-04

**Authors:** Collin J. Popp, Emily N. C. Manoogian, Blandine Laferrère

**Affiliations:** Department of Population Health, New York University Langone Health, Institute for Excellence in Health Equity, Center for Healthful Behavior Change, Grossman School of Medicine, New York University, New York, New York; Regulatory Biology Laboratory, Salk Institute for Biological Studies, La Jolla, California; Division of Endocrinology, Nutrition Obesity Research Center, Columbia University Irving Medical Center, New York, New York

Obesity is a serious health issue in the US, with a prevalence of 40% in adults.^1^ Calorie restriction through dietary modification remains the foundation of obesity management; however, traditional dietary modification through low-calorie diets is resource-heavy and shows poor long-term adherence, resulting in subsequent weight regain.^2^ Alternative lifestyle interventions include meal timing strategies, which consider the interplay between food intake and circadian biology. Meal timing strategies include time-restricted eating (TRE), meal frequency, and 24-hour caloric distribution. All of these are low-cost and easy-to-adopt approaches that may serve as treatment and prevention of metabolic diseases (eg, type 2 diabetes and hypertension).

In this systematic review and meta-analysis, Liu et al^3^ aimed to evaluate the existing evidence and determine the long-term (≥12 weeks) associations of meal timing strategies with anthropometrics and metabolic outcomes in adults with and without metabolic disease. Study selection included randomized clinical trials (RCTs) that enrolled adults aged 18 years or older, evaluated within-day meal timing patterns for 12 weeks or longer, and reported anthropometric measures. The main outcome was weight change (kilograms), and secondary outcomes included lean and/or fat-free mass, waist circumference, hemoglobin A_1c_ level, fasting glucose level, low-density lipoprotein level, systolic and diastolic blood pressure, and energy intake. A total of 29 RCTs were included, and a total of 2430 participants were enrolled, with a median follow-up duration of 12 weeks. The study populations were middle-aged, mostly female (69%), with obesity and a mean body mass index (BMI; calculated as weight in kilograms divided by height in meters squared) of 33. One-third of the studies were conducted in the US, 90% were parallel-group RCTs, and more than one-half of the recruited participants were from outpatient and/or community settings. Interventions that were evaluated included TRE (17 studies [59%], with 10 of them [59%] evaluating an 8-hour eating window), meal frequency (8 studies [28%]), and timing of caloric distribution over the biological day (4 studies [14%]).^3^

The main findings of the meta-analysis by Liu et al^3^ suggest meal timing strategies show modest changes in body weight, BMI, lean mass, and waist circumferences for interventions 12 weeks or longer. TRE had a modest effect on relative and absolute weight change (relative mean difference [MD], −1.37 kg; absolute MD, 1.82%), which is in line with previously published meta-analyses.^4,5^ Lower meal frequency was shown to have a slight reduction in body weight and BMI. Caloric distribution of energy intake earlier in the biological day resulted in a greater reduction in body weight, BMI, and waist circumference, which are supported by Young et al,^6^ who found that distributing energy intake earlier in the day, in the context of an energy-reduced diet, results in significantly great weight loss (MD, −1.23 kg).

Liu et al^3^ also examined the associations of meal timing strategies with metabolic outcomes. However, only TRE interventions resulted in significant outcomes, specifically on hemoglobin A_1c_, plasma glucose, and low-density lipoprotein levels and energy intake. The evidence was uncertain for the impact of meal frequency and caloric distribution. Overall, the association of meal timing strategies with both anthropometrics and metabolic outcomes was positive, and the authors note the overall quality of the evidence to be low as a result high risk of bias and heterogeneity. There was also significant heterogeneity of protocols, with some studies also incorporating other interventions that could impact results, such as exercise, caloric restriction, dietary counseling, protein shakes, and weighing food.

This systematic review and meta-analysis by Liu and colleagues^3^ highlights key gaps in the literature. More-precise methods for the assessment of diet, adherence to the intervention, body composition, and metabolic outcomes are needed in meal timing and/or TRE interventions. For example, Liu et al^3^ reported a slightly greater reduction in energy intake in TRE interventions; however, the majority of these interventions collected energy intake using self-reported methods (eg, 24-hour recalls) which are notoriously inaccurate.^7^ Self-reported methods in nutritional science research are a consistent limitation because of reporting bias and methodological inaccuracies. Most of the studies reported by Liu and colleagues^3^ assessed adherence with self-reporting. Monitoring of food intake in real-time with a smartphone application was done in a few of the referenced TRE studies and none of the meal timing or calorie distribution interventions. Future meal timing interventions may consider using objective measures to examine adherence, such as with continuous glucose monitors or wrist motion with actigraphy.^8^

In conclusion, Liu and colleagues^3^ support prior meta-analyses that meal timing strategies may be effective for weight management and metabolic outcomes. Although collectively these findings are positive, the overall effects are modest and their clinical application is questionable. Meal timing strategies may also be beneficial when paired with additional lifestyle modifications (eg, caloric restriction and physical activity). It is also important to note that aside from having elevated weight, most participants were otherwise healthy, which makes it unlikely to see any large changes in other health metrics. More research is needed to elucidate the impact of meal timing strategies, especially with meal frequency and caloric distribution, using more precise methods with long-term follow-up.
